# Delivery efficiency of an Elekta linac under gated operation

**DOI:** 10.1120/jacmp.v15i5.4713

**Published:** 2014-09-08

**Authors:** Guoqiang Cui, David J. Housley, Fan Chen, Vivek K. Mehta, David M. Shepard

**Affiliations:** ^1^ Department of Radiation Oncology Keck School of Medicine of the University of Southern California Los Angeles CA; ^2^ Department of Radiation Oncology Swedish Cancer Institute, Swedish Health Services Seattle WA USA

**Keywords:** delivery efficiency, beam‐on delay, volumetric modulated arc therapy, dosimetric accuracy, respiratory gating

## Abstract

In this study, we have characterized the efficiency of an Elekta linac in the delivery of gated radiotherapy. We have explored techniques to reduce the beam‐on delay and to improve the delivery efficiency, and have investigated the impact of frequent beam interruptions on the dosimetric accuracy of gated deliveries. A newly available gating interface was installed on an Elekta Synergy. Gating signals were generated using a surface mapping system in conjunction with a respiratory motion phantom. A series of gated deliveries were performed using volumetric modulated arc therapy (VMAT) treatment plans previously generated for lung cancer patients treated with stereotactic body radiotherapy. Baseline values were determined for the delivery times. The machine was then tuned in an effort to minimize beam‐on delays and improve delivery efficiency. After that process was completed, the dosimetric accuracy of the gated deliveries was evaluated by comparing the measured and the planned coronal dose distributions using gamma index analyses. Comparison of the gated and the non‐gated deliveries were also performed. The results demonstrated that, with the optimal machine settings, the average beam‐on delay was reduced to less than 0.22 s. High dosimetric accuracy was demonstrated with gamma index passing rates no lower than 99.0% for all tests (3%/3 mm criteria). Consequently, Elekta linacs can provide a practical solution for gated VMAT treatments with high dosimetric accuracy and only a moderate increase in the overall delivery time.

PACS numbers: 87.56.bd, 87.55.de, 87.55.ne

## I. INTRODUCTION

Complex radiotherapy delivery techniques, such as step‐and‐shoot intensity‐modulated radiotherapy (IMRT) and respiratory gated treatments, incorporate numerous beam interruptions that can negatively impact the overall delivery efficiency. In step‐and‐shoot IMRT, the beam is interrupted between segments and is held off while the multileaf collimator (MLC) leaves are advanced to their next positions.^1^, ^2^ When the beam is resumed, there is a time delay before the generation of the beam. In respiratory gated treatments, the beam is triggered on and off based on either the phase of the breathing cycle or the amplitude of the breathing depth.^3^, ^4^

There is a delay from the moment the patient enters the gating window until the instant that the beam begins to be delivered. While the individual time delays or beam‐on delays are modest in length, the cumulative impact of the delays can significantly increase overall delivery times, and has historically made respiratory gated delivery impractical on Elekta linacs. The beam‐on delays are particularly important for complicated delivery techniques, such as gated volumetric modulated arc therapy (VMAT), due to the fact that the delivery includes simultaneous variation of the MLC leaf speeds, dose rate, and gantry rotation.^5^, ^6^

Reducing the beam‐on delay and improving the overall delivery efficiency represents a challenge because this delay is impacted by numerous parameters within the linac control software, as well as the underlying design of the linac itself. Moreover, it is critical to verify that there is no loss in the dosimetric accuracy of the dose delivery due to incorporation of numerous beam interruptions.

The beam characteristics and dosimetric accuracy of gated beam delivery has been evaluated for Varian,^1^, ^7^ Siemens,^4^, ^8^ and Brainlab systems.^9^, ^10^ For Elekta linacs, gated delivery has focused on forced breath‐hold with the Active Breathing Coordinator (ABC) system.^(11)^ The first commercial gating interface for Elekta linacs has recently become available. Linac performance using this gating interface has been reported in terms of basic dosimetric accuracy, flatness, and symmetry.^(12)^ However, the beam‐on delay associated with each beam interruption and its impact on the delivery efficiency, as well as the dosimetric accuracy, have not been addressed. These are critical components of gating an Elekta linac, where beam‐on delays of up to 4 s have been previously reported.^(13)^

In this study, we characterized the delivery efficiency of VMAT with an Elekta linac under gated operation and investigated the effect of beam interruptions on the dosimetric accuracy of gated deliveries. We explored techniques to reduce the beam‐on delay and improve the delivery efficiency of gated VMAT. Three lung stereotactic body radiotherapy (SBRT) VMAT plans were delivered under gated operation. The dosimetric accuracy was evaluated by comparing the measured and the planned coronal dose distributions using gamma index analyses. Comparison of the gated and the non‐gated deliveries were also performed.

## II. MATERIALS AND METHODS

### A. The Elekta Response gating control interface

**Figure 1:**
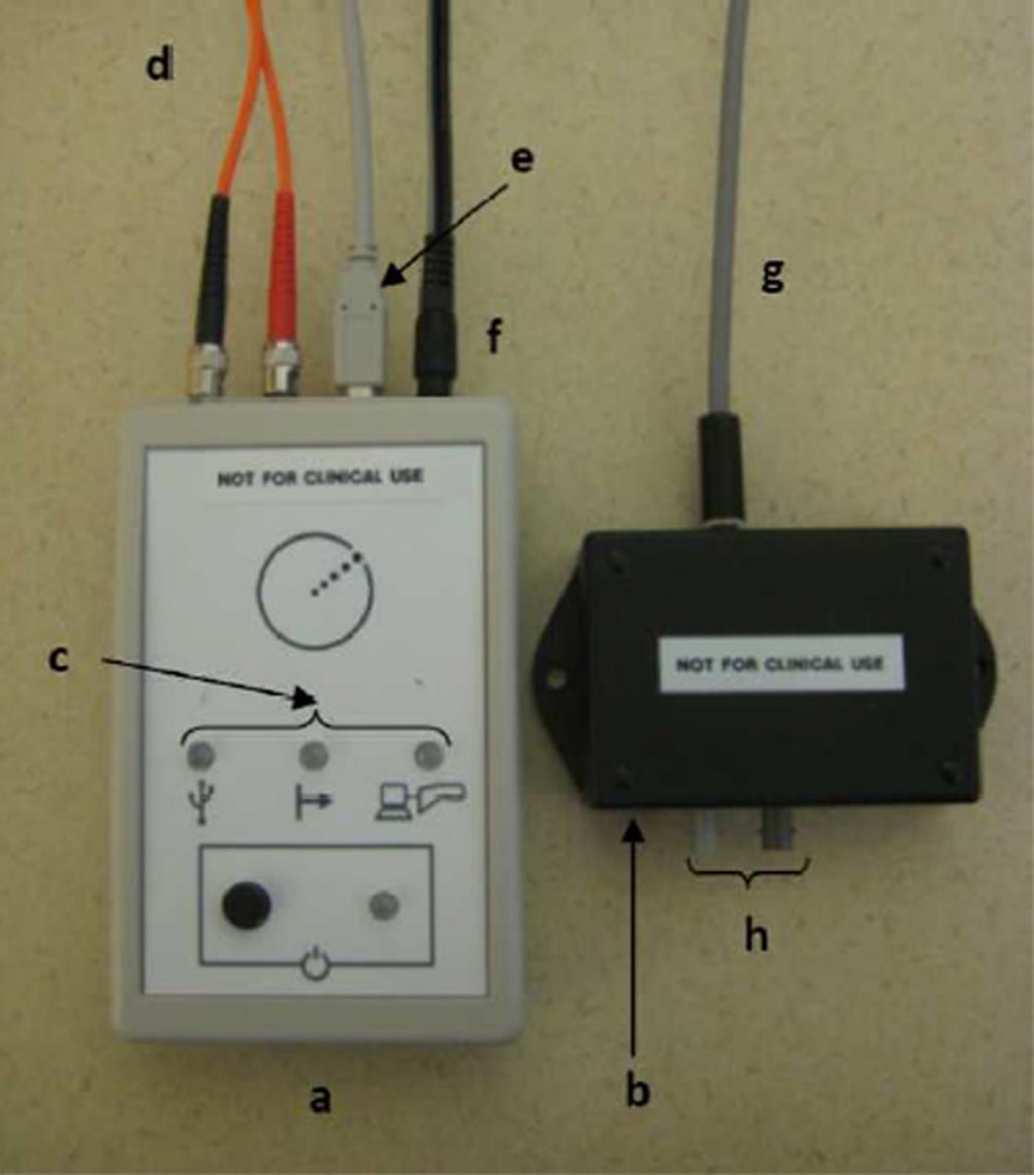
Elekta Response gating control interface: a=gating switch box; b=relay module; c=LEDs; d=duplex fiber optic cable, which connects the gating switch box and the relay module; e=USB cable for gating signal input; f=power supply; g=relay‐PRF cable, which connects directly to the PRF EN chain of the Elekta linac; h=duplex fiber optic cable connectors.

The Elekta Response gating control interface (Elekta AB, Stockholm, Sweden) allows any third‐party system to pause and resume the delivery of radiation by controlling physical relays that interrupt the pulse repetition frequency enable (PRF EN) signal during the beam‐off period. A preclinical version of the Elekta Response gating control interface was installed on an Elekta Synergy in our clinic. The Response consists of two main components: the gating switch box and the relay module (see Fig. 1). The gating switch box allows the user to manually enable and disable the gating system. It also presents information to the user through the illumination of light‐emitting diodes (LEDs). It communicates to the computer that controls the gating via a universal serial bus (USB) and instructs the relay module when to open or close the signal relays.

The gating switch box is placed in the treatment console area, while the relay module is placed in the interface cabinet control area in the treatment room. The two components are connected via a duplex fiber optic cable. The relay module is responsible for opening or closing physical relays that will interrupt the PRF chain of the Elekta linac. The minimum system configuration required to use the Response gating control interface is a fast tuning magnetron and a linac control system with Desktop version 6.0 or higher.^(13)^

### B. Monitoring breathing motion and generating the gating signal

**Figure 2:**
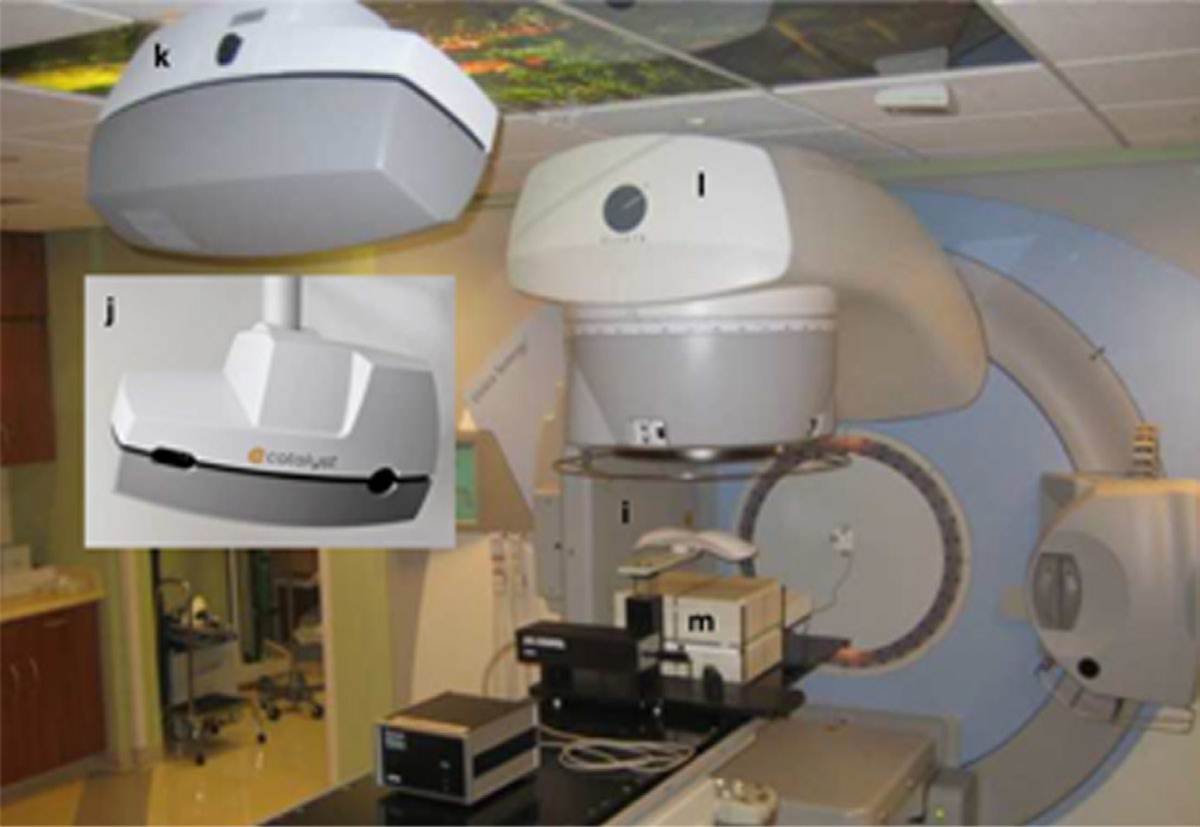
Experimental setup for the gating test: i= the CIRS dynamic thorax phantom with a chest plate on the breathing platform; j and k= the front and rear views of the C‐RAD Catalyst camera system; l=the Elekta Synergy linac; m= the IBA MatriXX Evolution with MultiCube and gantry angle sensor.

Breathing motion can be monitored either directly by imaging the target^(14)^ or indirectly by using surrogates such as fiducial markers,^(15)^ abdominal/chest surface motion,^16^, ^17^, ^18^ or spirometry.^(19)^ In this study, surface motion was used as a surrogate for breathing motion. The experimental setup is shown in Fig. 2. Surface motion was simulated using a moving chest plate mounted on the breathing platform of the CIRS dynamic thorax phantom (CIRS Inc., Norfolk, VA), as indicated by item *i* in Fig. 2. The chest plate motion was programmed with a constant amplitude range of ±3mm and a period of 4 sec using a $\text{cos}{\;}^{6}\omega \;t$ waveform, approximating nominal breathing patterns.

The motion of the chest plate was captured by a surface mapping system, the C‐RAD Catalyst (C‐RAD AB, Uppsala, Sweden).^(20)^ The Catalyst employs three high‐power LEDs (blue, green, and red) to project light patterns onto the object surface. The blue light is used for surface imaging and is detected by a monochrome charge‐coupled device (CCD) camera with a frame rate of 202 frames per sec. The green and red lights project surface mismatches (actual vs. reference image) onto the area where the mismatch is detected for motion monitoring and patient positioning. Items *j* and *k* in Fig. 2 show the front and rear views of the Catalyst camera system. The captured motion was verified to have the same amplitude range and period as the programmed chest plate motion with undetectable phase delay. The gating was based on amplitude and the thresholds can be set within the Catalyst system. The gating signal was then input into the gating switch box via the USB connection.

### C. Beam deliveries and data collection and analyses

All radiation deliveries were carried out on an Elekta Synergy linac. Treatment plans from three lung patients previously treated with non‐gated SBRT were used for this evaluation. The prescribed dose for each plan was 12 Gy per fraction for 4 fractions. The plans were created in the Monaco treatment planning system ver. 3.0 (Elekta AB). For each case, one non‐gated and two gated deliveries were delivered to a static ion chamber array inserted into plastic water phantom, the IBA MatriXX Evolution with the MultiCube phantom (IBA Dosimetry GmbH, Schwarzenbruck, Germany), item *m* as shown in Fig. 2. Two gating windows (GWs), 77% and 66%, centered on the end of exhalation were used for the gated deliveries. For each delivery, we measured the actual delivery time between the two time points of the initial beam on and the end of the plan delivery. As a reference, we calculated an ideal delivery time assuming no time delay in the entire system, for the gated deliveries, as the actual non‐gated delivery time divided by the gating window in Eq. (1):

(1) $$\text{Ideal delivery time}=\frac{\text{Non}‐\text{gated delivery time}}{\text{Gating window}}$$

Baseline values of the actual delivery times were established with a binned dose rate (BDR) of 8 dose‐rate levels and a default accelerator gun hold‐on time (GHT) of 1.38 s. The BDR allows the dose rate to be reduced by factors of 2 from the maximum dose rate of 600 MU/min to zero.^21^, ^22^ The accelerator gun hold‐on time is the time that the electron gun will remain at its last active value prior to the beam interruption. After the beam is held off for a period longer than the gun hold‐on time, the accelerator gun switches to standby mode. Consequently it takes longer for the gun to come back to an active mode and stabilize from the standby mode than from a previously active mode prior to the beam interruption.

Ideal delivery times of the gated deliveries were calculated using Eq. (1) to compare with the corresponding actual delivery times. The differences between the actual delivery times and the ideal delivery times are presumably caused by the beam‐on delays after each beam interruption. Improving the delivery efficiency can best be characterized by looking at the reduction in the average beam‐on delay of single breathing cycles. We used Eq. (2) to calculate the average beam‐on delay. Of note is that with this method, the average beam‐on delay represents the total time delay of the entire system, including the delays in the Catalyst system, the Response, and the linac delivery system.

(2) $$\text{Average beam on delay}=\frac{\text{Actual delivery time}‐\text{Ideal delivery time}}{\text{Number of breathing cycles of the gated delivery}}$$

In an effort to mitigate the impact of the radiation dose‐rate ramp‐up and stabilization after each beam interruption, we upgraded the linac to a new control system. The new control software Integrity R1.1 (Elekta AB) includes a continuously variable dose rate (CVDR) of 256 dose‐rate levels for dynamic deliveries such as VMAT.^21^, ^22^ We repeated all the measurements with the CVDR. The delivery times and the average beam‐on delays between the deliveries with the BDR and the CVDR were then compared. The percent reduction in the delivery time was calculated by the difference between the actual and baseline delivery times divided by the baseline delivery time.

Another key development in further improving the delivery efficiency was increasing the accelerator gun hold‐on time. The default gun hold‐on time of 1.38 s proved too short for free‐breathing respiratory gating. This means that the accelerator gun went to a standby mode after the beam was held off for 1.38 s. When resuming the beam, it takes time for the gun current to come back and stabilize. The accelerator gun hold‐on time was adjusted to the maximum allowable value of 6.50 s. Once this adjustment was combined with the CVDR, further comparisons of the gated deliveries between two gun hold‐on times of 1.38 s and 6.50 s were performed.

Both gated and non‐gated deliveries were measured using a MatriXX Evolution as a means of evaluating the impact of the beam interruptions and the beam‐on delay on the dosimetric accuracy of the gated deliveries. The coronal dose distributions were compared using the Omnipro‐I'mRT software (IBA Dosimetry GmbH). The dosimetric accuracy of gated VMAT deliveries was determined using gamma index analyses. The gamma method in Omnipro‐I'mRT uses the same technique as reported by Low et al.^(23)^ For consistency, the selected region of interest (ROI) for gamma index analyses was a rectangle of $23.6\;\times \;10.0\;{cm}^{2}$ (23,937 pixels included) centered at the plan isocenter, which fully covered the targets in both the lateral direction (X direction) and the superior–inferior direction (Y direction). For comparison of the measured and the planned coronal dose distributions, we used a 3%/3 mm passing rate criteria, based on our clinical standards for quality assurance of IMRT and VMAT plans. For comparison of measurements with the gated and the non‐gated deliveries, we used a more stringent 2%/1 mm passing rate criteria, as the distance to agreement (DTA) should be minimal because the gated and the non‐gated plans were delivered using the same setup.

## III. RESULTS

### A. Baseline values of delivery times and average beam‐on delays

Table 1 shows the baseline values of the delivery times and the average beam‐on delays for three VMAT plan deliveries with the BDR and the default gun hold‐on time of 1.38 s. The breathing period of the surrogate motion was 4 s.

The baseline values of the actual delivery times of the two gated deliveries were substantially longer than the ideal delivery times by as much as 74.8%, as indicated by the delivery with the 66% gating window for Patient 3. The average beam‐on delays of the gated deliveries ranged between 1.00 s and 1.71 s. The smaller gating window (66% vs. 77%) led to larger beam‐on delays. It would not be practical to implement gated free‐breathing treatments with the BDR and the default GHT. As an example, when we used a 4 s breathing cycle in this test and a 25% gating window, the Synergy failed to deliver any radiation because the beam‐on delay was longer than the beam‐on time the phantom was in the gating window (1 s). In other words, the motion surrogate would exit the gating window before the beam irradiation would commence.

**Table 1:** This table shows the baseline values of the delivery times and the average beam‐on delays for three VMAT plan deliveries with the BDR and the default gun hold‐on time of 1.38 s. Column 1 is the patient and corresponding plan information. Column 2 is the gating window (the 100% gating window means non‐gated beam delivery). Columns 3, 4, and 5 are baseline values of the actual delivery time, ideal delivery time, and the average beam‐on delay

*Patient & Plan Info*.	*Gating Window*	*Actual Delivery Time (min)*	*Ideal Delivery Time (min)*	*Average Beam‐On Delay (s)*
1	100%	5.65	5.65	NA
One 360° arc,	77%	9.97	7.34	1.06
12 Gy, 2417 MUs	66%	14.22	8.56	1.59
2	100%	5.32	5.32	NA
One 180° arc,	77%	9.20	6.91	1.00
12 Gy, 2101 MUs	66%	13.80	8.06	1.66
3	100%	4.58	4.58	NA
One 180° arc,	77%	8.13	5.95	1.07
12 Gy, 1854 MUs	66%	12.13	6.94	1.71

### B. Improved delivery efficiency with continuously variable dose rate

Table 2 summarizes the delivery time, percent reduction in the delivery time, and the average beam‐on delay for all tests.

After switching from the BDR to the CVDR and maintaining the default GHT of 1.38 s, the average reductions in the delivery time were 8.0%, 28.2%, and 15.8% for non‐gated delivery and gated deliveries with 77% and 66% gating windows, respectively. The average beam‐on delays for 77% gating window were reduced to less than 0.22 s. However, the beam‐on delays for the 66% gating window did not decrease much for all three plans, as highlighted in blue in column 5. This was primarily due to the fact that with a 4 s breathing period, the beam‐off time for a 66% gating window was 1.36 s. Consequently, the accelerator gun would return to the standby mode with the default GHT of 1.38 s. When resuming the beam, it required time for the gun to come back to a stable mode to generate beam.

After increasing the GHT to the maximum allowable value of 6.50 s while maintaining the CVDR, the delivery times for the non‐gated and 77% gating window deliveries were reduced slightly by an average of 1.6% and 2.4%, respectively, as compared to previous tests with the default GHT of 1.38 s. But for the 66% gating window, the average reduction in the delivery time was 29.5% as compared to previous tests. Looking at the average beam‐on delays for the 66% gating window, they were also reduced to less than 0.22 s, just as those for 77% gating window in the previous test. Data are highlighted in red in column 5. This test verified that the GHT needs to be longer than the beam‐hold time during beam interruption to maintain its last active value for quickly turning the beam on.

To this end, for all the gated VMAT deliveries with the CVDR along with the maximum allowable accelerator gun hold‐on time of 6.50 s, the average reduction in the delivery time was 37.9% (range 29.0%–47.2%), as compared to the baseline values.

**Table 2:** This table summarizes the delivery time, percent reduction in the delivery time, and the average beam‐on delay for all tests. Column 1 is the patient and corresponding plan information. Column 2 is the gating window (the 100% gating window means non‐gated beam delivery). Columns 3, 4, and 5 are the actual delivery time, percent reduction in the delivery time, and the average beam‐on delay for all tests. Baseline data are listed in bold, with the BDR and the gun hold‐on time (GHT) of 1.38 s. Results of the improved results, as against the baseline values, using the CVDR with GHTs=1.38s and 6.50 s are listed in parenthesis and brackets, respectively

*Patient & Plan Info*.	*Gating Window*	*Actual Delivery Time (min)*	*Percent Reduction in the Delivery Time (%)*	*Average Beam‐on Delay (s)*
		BDR GHT=1.38s	CVDR GHT=1.38s	CVDR GHT=6.50s	CVDR GHT=1.38s	CVDR GHT=6.50s	BDR GHT=1.38s	CVDR GHT=1.38s	CVDR GHT=6.50s
1	100%	**5.65**;	(5.42);	[5.15]	(4.07);	[8.85]	**NA**		
One 360° arc,	77%	**9.97**;	(7.45);	[7.08]	(25.28);	[28.99]	**1.06**;	(0.22);	[0.22]
12 Gy, 2417 MUs	66%	**14.22**;	(12.52);	[8.13]	(11.95);	[42.83]	**1.59**;	(1.38);	[0.16]
2	100%	**5.32**;	(4.80);	[4.80]	(9.77);	[9.77]	**NA**		
One 180° arc,	77%	**9.20**;	(6.56);	[6.40]	(28.70);	[30.43]	**1.00**;	(0.20);	[0.10]
12 Gy, 2101 MUs	66%	**13.80**;	(11.38);	[7.47]	(17.54);	[45.87]	**1.66**;	(1.44);	[0.11]
3	100%	**4.58**;	(4.12);	[4.12]	(10.04);	[10.04]	**NA**		
One 180° arc,	77%	**8.13**;	(5.65);	[5.52]	(30.50);	[32.10]	**1.07**;	(0.21);	[0.12]
12 Gy, 1854 MUs	66%	**12.13**;	(9.95);	[6.40]	(17.97);	[47.24]	**1.71**;	(1.49);	[0.10]

### C. Dosimetric accuracy of VMAT plans under gated operation

**Figure 3:**
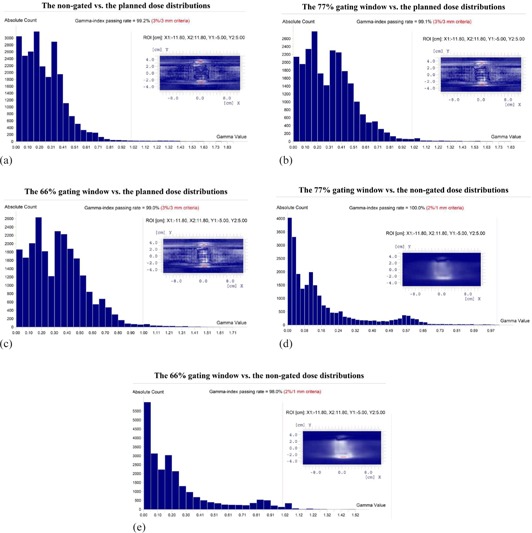
Gamma index analyses of Patient 1's coronal dose distributions: (a)–(c) the measurements of the non‐gated, 77% and 66% gating windows deliveries vs. the planned, respectively (3%/3 mm criteria); (d)–(e) the gated 77% and 66% gating windows vs. the non‐gated deliveries, respectively (2%/1 mm criteria). The horizontal axis is the gamma value. The red vertical line indicates where the gamma value is equal to 1. The vertical axis is the absolute count of the number of the pixels in the corresponding gamma range. The insets show the difference in the dose/DTA of the compared dose distributions in the selected ROI. The blue, white, and red color scales represent that the gamma value <1, ∼1, and >1, respectively. These data were collected with the CVDR and the default accelerator GHT of 1.38 s.

The dosimetric accuracy of gated VMAT deliveries was evaluated by comparing coronal dose distributions using gamma index analyses. For comparisons of the measured and the planned coronal dose distributions, we used the 3%/3 mm criteria, based on our clinical standards for quality assurance of IMRT and VMAT plans. The passing rates for all tests of three VMAT plans were no lower than 99.0%. Figures 3(a)–3(c) show examples (Patient 1) of the comparison of the measured and the planned coronal dose distributions using gamma index analyses.

Comparison of measurements with the gated and the non‐gated deliveries were also performed. Using the 3%/3 mm criteria, the gamma index passing rates for all tests were 100%. When using the more stringent 2%/1 mm criteria, for the gated delivery with the 77% gating window, the gamma index passing rates were still all 100%. For the gated delivery with the 66% gating window, the passing rate was higher than 97.0% for all tests. The use of narrower gating window results in more beam interruptions for the plan delivery and can lead to a lower passing rate. Figures 3(d) and 3(e) show the comparison of the measurements with the gated and the non‐gated deliveries for the same patient.

## IV. DISCUSSION

For VMAT deliveries using an Elekta Synergy under gated operation with the CVDR and the maximum allowable accelerator GHT of 6.50 s, the delivery time was reduced by an average of 37.9% and the average beam‐on delay was reduced to less than 0.22 s. Consequently, gated treatment plans can be delivered in an efficient manner. High dosimetric accuracy was demonstrated with gamma index passing rates that were no lower than 99.0% for all tests (3%/3 mm criteria). Of note is that the dosimetric accuracy evaluation in this study was based on comparison of planar dose distributions, which is an integrated dose comparison. For detailed beam characteristics, including pulse measurements, please refer to a report by Evans et al.^(12)^

In this study, the delivery verifications were performed using a static phantom. A static phantom was used because the goal was to demonstrate that the incorporation of numerous beam interruptions in a gated beam delivery does not impact the accuracy of the delivered dose distribution on an Elekta linac. While a moving phantom would provide a more complete end‐to‐end test, the focus of this study was on characterizing machine performance.

The improved results were measured based on dynamic deliveries, such as VMAT, because the CVDR works well for dynamic deliveries. For gated nondynamic deliveries, the beam‐on delay after beam interruption still greatly impacts the delivery time. Moreover, the CVDR is only available in Elekta linac control system Integrity R1.1 or higher versions; and in order to use the Response gating control interface, the linac requires a fast tuning magnetron.^(13)^ For consistency and simplicity, the surface motion we used as a surrogate for breathing motion has a constant amplitude range of ± 3 mm and a period of 4 s. For future study, different breathing periods with different gating windows can be tested; but this investigation focused on preliminary testing of the first commercial gating solution for Elekta linacs.

## V. CONCLUSIONS

We have characterized the efficiency and delivery of an Elekta Synergy under gated operation. With optimized performance, the average beam‐on delay was reduced to less than 0.22 s. We also investigated the impact of frequent beam interruptions (due to respiratory gating) on the dosimetric accuracy and precision of the linac system. We have demonstrated that Elekta linacs can efficiently deliver complex treatment plans, such as gated VMAT, with high dosimetric accuracy. The results should offer a baseline reference for Elekta users.

## ACKNOWLEDGMENTS

This work was supported by a research grant from Elekta. The authors thank Sven Sund of Elekta for useful discussions and help with tuning our linacs for optimal delivery.

## Supporting information

Supplementary Material
