# Supplementary figures and images for: Delivery efficiency of an Elekta linac under gated operation
Source: J Appl Clin Med Phys. 2014 Sep 8;15(5):2–11. doi: 10.1120/jacmp.v15i5.4713 (PMC5711085; doi:10.1120/jacmp.v15i5.4713)

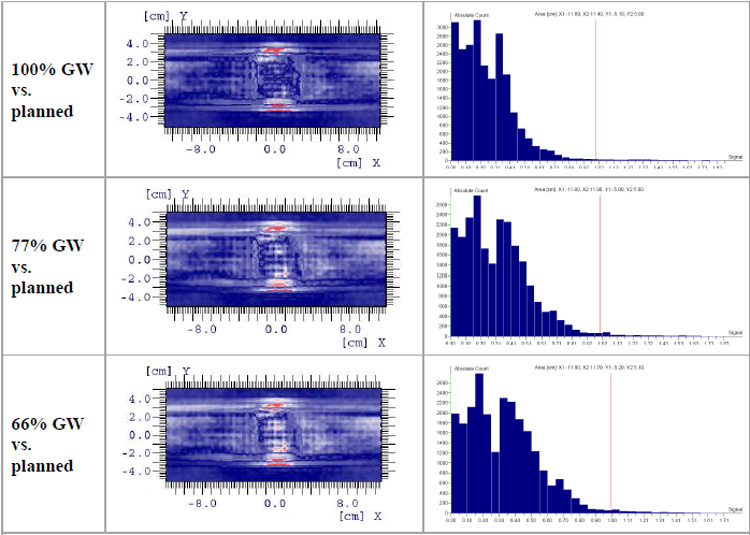

Supplement: Supplementary file 1 — Supplementary Material [file ACM2-15-002-s001.JPG]
